# Dexamethasone restores odontogenic differentiation in lipopolysaccharide-inflamed human dental pulp stem cells

**DOI:** 10.1590/1807-3107bor-2026.vol40.093

**Published:** 2026-11-09

**Authors:** Yong DU, Yang YANG, Hongguang LIAN, Haiyan YANG, Haobo LI, Dongmei CHENG, Han GUO, Zhiyong ZHANG

**Affiliations:** (a) The Second Hospital of Hebei Medical University, Department of Oral Medicine, Shijiazhuang, Hebei, China.; (b) The Second Hospital of Hebei Medical University, Department of Pathology, Shijiazhuang, Hebei, China

**Keywords:** Dexamethasone, LipopolysaccharideS, Pulpitis

## Abstract

Pulpitis caused by bacterial infection compromises dental pulp regeneration. This study investigated whether dexamethasone (Dex) could restore odontogenic differentiation in lipopolysaccharide (LPS)-inflamed human dental pulp stem cells (hDPSCs) and examined the involvement of TLR4/NF-κB signaling. hDPSCs isolated from healthy third molars were characterized by flow cytometry, and Cell Counting Kit-8 assays were used to identify noncytotoxic concentrations of LPS (2 μg/mL) and Dex (10^−8^ mol/L). In an LPS-induced inflammatory model, with or without Dex pretreatment, the expression and secretion of proinflammatory cytokines (IL-6, TNF-α, and IL-1β) were quantified by quantitative real-time polymerase chain reaction (qRT-PCR) and enzyme-linked immunosorbent assay (ELISA). TLR4/NF-κB activation was evaluated by qRT-PCR and western blotting, including measurement of total and phosphorylated p65. Odontogenic differentiation was assessed using alkaline phosphatase (ALP) activity and Alizarin Red S (ARS) mineralization assays. Data were analyzed by one-way analysis of variance followed by Tukey’s post hoc test. LPS markedly increased cytokine expression and secretion, upregulated TLR4, increased the p-p65/p65 ratio, and reduced ALP activity and mineralized nodule formation. Dex pretreatment significantly attenuated cytokine induction, reduced TLR4 expression and p65 phosphorylation, and restored ALP activity and mineralization relative to LPS alone. Low-dose Dex attenuated LPS-induced inflammation in hDPSCs by suppressing the TLR4/NF-κB axis and restored odontogenic differentiation. These findings support further investigation of localized Dex delivery as an adjunct to vital pulp therapy.

## Introduction

Pulpitis is a common and often painful inflammatory condition in endodontics that is primarily initiated by microbial invasion after dental caries, trauma, or restorative procedures.^1^ Because the dental pulp is enclosed within a rigid dentin chamber, its capacity for immune defense and repair is limited. If inflammation is not controlled, it may progress to pulp necrosis and periapical disease, necessitating root canal treatment or tooth extraction and resulting in loss of tooth vitality.^2^ Accordingly, vital pulp therapy (VPT) has emerged as an important alternative to symptom-oriented treatment because it aims to preserve pulp vitality and promote tissue regeneration.^3^ Human dental pulp stem cells (hDPSCs), a population of mesenchymal stromal cells (MSCs) located in the perivascular niche, are central to this process. These cells possess self-renewal capacity and multilineage differentiation potential, including differentiation toward odontoblast-like cells required for dentin bridge formation.^4,5^ However, the inflammatory microenvironment can substantially impair the regenerative capacity of hDPSCs and shift cellular activity from tissue repair toward immune defense.^6^ Therapeutic strategies that simultaneously limit excessive inflammation and preserve or restore the differentiation capacity of hDPSCs are therefore needed.

Lipopolysaccharide (LPS), a major constituent of the outer membrane of Gram-negative bacteria, is an important virulence factor in pulpitis.^7^ LPS activates innate immune signaling by binding to Toll-like receptor 4 (TLR4), a pattern-recognition receptor expressed by hDPSCs, and initiating a predominantly TLR4/MyD88-dependent cascade.^8,9^ A major downstream effector of this cascade is nuclear factor kappa B (NF-κB). Following stimulation, inhibitor of κB (IκB) is phosphorylated and degraded, permitting translocation of the NF-κB p65 subunit to the nucleus.^10,11^ Nuclear p65 promotes transcription of proinflammatory cytokines, including interleukin 6 (IL-6), tumor necrosis factor alpha (TNF-α), and interleukin 1 beta (IL-1β).^12^ Although this response contributes to acute host defense, sustained or excessive TLR4/NF-κB activation promotes tissue injury and impairs odontogenic differentiation, including ALP activity and mineralized matrix deposition.^13^ The TLR4/NF-κB axis is therefore a plausible therapeutic target for modulating pulpal inflammation.

Glucocorticoids are potent anti-inflammatory and immunosuppressive agents. Dexamethasone (Dex), a synthetic glucocorticoid, has been used in dental applications because of its ability to reduce pain and edema.^14-17^ Dex suppresses inflammatory mediator production in several cell types, including human lung fibroblasts,^18^ and its anti-inflammatory activity is frequently associated with inhibition of NF-κB signaling.^19^ Dex is also a standard component of osteogenic induction media, and transcriptomic analyses have linked it to mineralization-related gene expression in human dental pulp cells.^20^ Nevertheless, its effects on hDPSCs exposed to a substantial inflammatory challenge remain incompletely characterized. Wang et al. showed that the corticosteroid betamethasone suppressed LPS-induced inflammation in dental pulp cells through NF-κB inhibition,^21^ however, it remains unclear whether Dex can restore odontogenic differentiation compromised by LPS rather than merely reducing cytokine production. Clarifying this inflammation-regeneration balance is important for evaluating the potential role of Dex in regenerative endodontics.

We hypothesized that Dex would exert a dual protective effect in hDPSCs by attenuating LPS-induced inflammation and restoring impaired odontogenic differentiation. We established an *in vitro* inflammatory model using LPS-stimulated hDPSCs and evaluated cytokine expression and secretion at the mRNA and protein levels. We also investigated TLR4/NF-κB signaling and assessed functional recovery using ALP activity and mineralization assays. The study was reported in accordance with the Checklist for Reporting *In Vitro* Studies (CRIS) guidelines.^22^

## Methods

### Ethical approval and cell isolation

The study was approved by the Ethics Committee of The Second Hospital of Hebei Medical University (approval no. 2025-R649). Healthy third molars were obtained from adults aged 18–25 years who underwent extraction for orthodontic indications or impaction at the Department of Oral and Maxillofacial Surgery. Written informed consent was obtained from all donors before sample collection. hDPSCs were isolated using a tissue-explant enzymatic digestion method, as previously described by Gronthos et al.^23^ Briefly, pulp tissue was removed aseptically, minced into 1-mm^3^ fragments, and digested with 3 mg/mL collagenase type I (Gibco, USA) and 4 mg/mL dispase (Sigma-Aldrich, USA) for 40 min at 37°C.^23^ The cell suspension was centrifuged and resuspended in Dulbecco’s modified Eagle medium (DMEM; Gibco) supplemented with 10% fetal bovine serum (FBS; Gibco) and 1% penicillin-streptomycin (Solarbio, China). Cells were maintained at 37°C in a humidified atmosphere containing 5% CO_2_. Cells at passages 3-5 were used in all experiments.

### Flow cytometric characterization

To confirm the mesenchymal stromal cell phenotype, passage 3 hDPSCs were trypsinized, washed with phosphate-buffered saline, and incubated for 30 min at 4 °C in the dark with fluorescein isothiocyanate- or phycoerythrin-conjugated monoclonal antibodies against CD90, CD105, CD34, and CD45 (BD Biosciences, San Jose, USA). Nonspecific isotype-matched IgG antibodies served as negative controls. Surface-marker expression was analyzed using a flow cytometer (Beckman Coulter, Brea, USA).

### Cell viability assay

Cell viability was assessed using the Cell Counting Kit-8 (CCK-8; Sigma-Aldrich, St. Louis, USA). hDPSCs were seeded in 96-well plates at 5 × 10^3^ cells/well and allowed to adhere for 24 h. The cells were then exposed for 24 h to graded concentrations of Dex (10^9^-10^5^ mol/L) or LPS (0.5-10 μg/mL). Subsequently, 10 μL of CCK-8 reagent was added to each well, and the cells were incubated for an additional 2 h. Absorbance was measured at 450 nm using a microplate reader (Thermo Fisher Scientific, Waltham, USA). Concentrations were selected for subsequent experiments by balancing experimental relevance with cell viability; prespecified thresholds were >90% viability for Dex and >80% viability for LPS in the nonlethal inflammatory model.

### Experimental design and treatment

hDPSCs were seeded in six-well plates or other culture vessels appropriate for each assay. Four experimental groups were established:

- Control group: cells cultured in complete medium alone.
- LPS group: cells stimulated with 2 μg/mL LPS (*Escherichia coli* O111:B4; Sigma) for 24 h.
- Dex + LPS group: cells pretreated with 10^8^ mol/L Dex (Sigma) for 1 h before the addition of 2 μg/mL LPS and then co-incubated for 24 h.
- Dex-alone group: cells treated with 10^8^ mol/L Dex for 24 h under otherwise identical culture conditions.

The Dex-alone group was included to determine whether low-dose Dex altered basal inflammatory or odontogenic parameters in the absence of LPS.

### Quantitative real-time polymerase chain reaction

Total RNA was extracted using TRIzol reagent (Vazyme, Nanjing, China), and RNA concentration and purity were assessed with a NanoDrop spectrophotometer (Thermo Fisher Scientific). Complementary DNA was synthesized using HiScript III RT SuperMix (Vazyme). Quantitative real-time polymerase chain reaction (qRT-PCR) was performed using ChamQ Universal SYBR qPCR Master Mix (Vazyme) on an ABI 7500 Real-Time PCR System. The cycling conditions were 95 °C for 30 s, followed by 40 cycles of 95°C for 10 s and 60°C for 30 s. Relative gene expression was calculated using the 2^−^ΔΔ^Ct^ method, with *GAPDH* as the reference gene. Primer sequences are provided in Table 1.

**Table 1:** Primer sequences used for qRT-PCR.

Gene	Forward primer (5′-3′)	Reverse primer (5′-3′)
*IL-6*	ACTCACCTCTTCAGAACGAATTG	CCATCTTTGGAAGGTTCAGGTTG
*TNF-*α	CCTCTCTCTAATCAGCCCTCTG	GAGGACCTGGGAGTAGATGAG
*IL-1*β	ATGATGGCTTATTACAGTGGCAA	GTCGGAGATTCGTAGCTGGA
*TLR4*	AGACCTGTCCCTGAACCCTAT	CGATGGACTTCTAAACCAGCCA
*NFKB1*	TGTGGAGACGCTGAGGAGCAC	GGGAAGACATGGATGACCTGAG
*GAPDH*	GGAGCGAGATCCCTCCAAAAT	GGCTGTTGTCATACTTCTCATGG

### Enzyme-linked immunosorbent assay

Cell-culture supernatants were collected after 24 h of treatment. Concentrations of IL-6, TNF-α, and IL-1β were measured using human-specific enzyme-linked immunosorbent assay kits (R&D Systems, Minneapolis, USA) according to the manufacturer’s instructions. Absorbance was measured at 450 nm.

### Western blot analysis

Total protein was extracted using radioimmunoprecipitation assay buffer containing protease and phosphatase inhibitors (Servicebio, Wuhan, China), and protein concentration was determined using a bicinchoninic acid assay. Equal amounts of protein (30 μg per lane) were separated by 10% sodium dodecyl sulfate-polyacrylamide gel electrophoresis and transferred to polyvinylidene difluoride membranes (Millipore, USA). The membranes were blocked with 5% nonfat milk and incubated overnight at 4°C with primary antibodies against TLR4 (1:1,000; Abcam), NF-κB p65 (1:1,000; Cell Signaling Technology [CST]), phospho-NF-κB p65 (Ser536; 1:1,000; CST), IL-1β (1:1,000; Abcam), and β-actin (1:5,000; Servicebio). After washing with Tris-buffered saline containing Tween 20, the membranes were incubated with horseradish peroxidase-conjugated secondary antibodies (1:5,000) for 1 h. Protein bands were visualized using enhanced chemiluminescence reagent and quantified with ImageJ software.

### Alkaline phosphatase activity and staining

For functional assays, cells were cultured in osteogenic induction medium (OM) under the specified treatment conditions. The OM consisted of DMEM supplemented with 10% FBS, 1% penicillin-streptomycin, 50 μg/mL L-ascorbic acid 2-phosphate (Sigma-Aldrich), and 10 mmol/L β-glycerophosphate (Sigma-Aldrich). Dexamethasone was excluded from the basal OM to avoid confounding the experimental comparison. After 7 days, cells were lysed, and ALP activity was measured using an ALP Assay Kit (Beyotime, China) and normalized to total protein content. ALP staining was performed using the BCIP/NBT Alkaline Phosphatase Color Development Kit (Beyotime) according to the manufacturer’s protocol.

### Alizarin red S staining

To assess mineralization, hDPSCs were cultured in OM under the specified treatment conditions for 21 days. Cells were fixed with 4% paraformaldehyde and stained with 2% Alizarin Red S (pH 4.2; Sigma) for 20 min. For quantitative analysis, the stain was solubilized with 10% cetylpyridinium chloride, and absorbance was measured at 562 nm.

### Statistical Analysis

All experiments were performed in triplicate. Data are presented as the mean ± standard deviation (SD). Statistical analyses were conducted using GraphPad Prism version 9.0 (GraphPad Software, Boston, USA). Differences among groups were evaluated by one-way analysis of variance followed by Tukey’s post hoc multiple-comparison test. Baseline cellular parameters were compared between the control and Dex-alone groups using a two-sided unpaired Student *t* test. A two-sided p-value < 0.05 was considered statistically significant.

## Results

### Characterization of hDPSCs and surface-marker expression

Primary hDPSCs migrated from pulp-tissue explants within 3-5 days and exhibited the characteristic spindle-shaped, fibroblast-like morphology (Figure 1A). After purification and passaging, the cells displayed a homogeneous morphology (Figure 1B). Flow cytometry was performed to confirm their mesenchymal stromal cell phenotype. As shown in Figure 1C, the cells strongly expressed CD90 (98.5% ± 1.2%) and CD105 (97.8% ± 1.5%) but showed minimal expression of the hematopoietic markers CD34 (1.2% ± 0.5%) and CD45 (0.8% ± 0.3%). This profile met the minimal phenotypic criteria established by the International Society for Cellular Therapy and confirmed the successful isolation of a highly enriched hDPSC population.^24^

**Figure 1:**
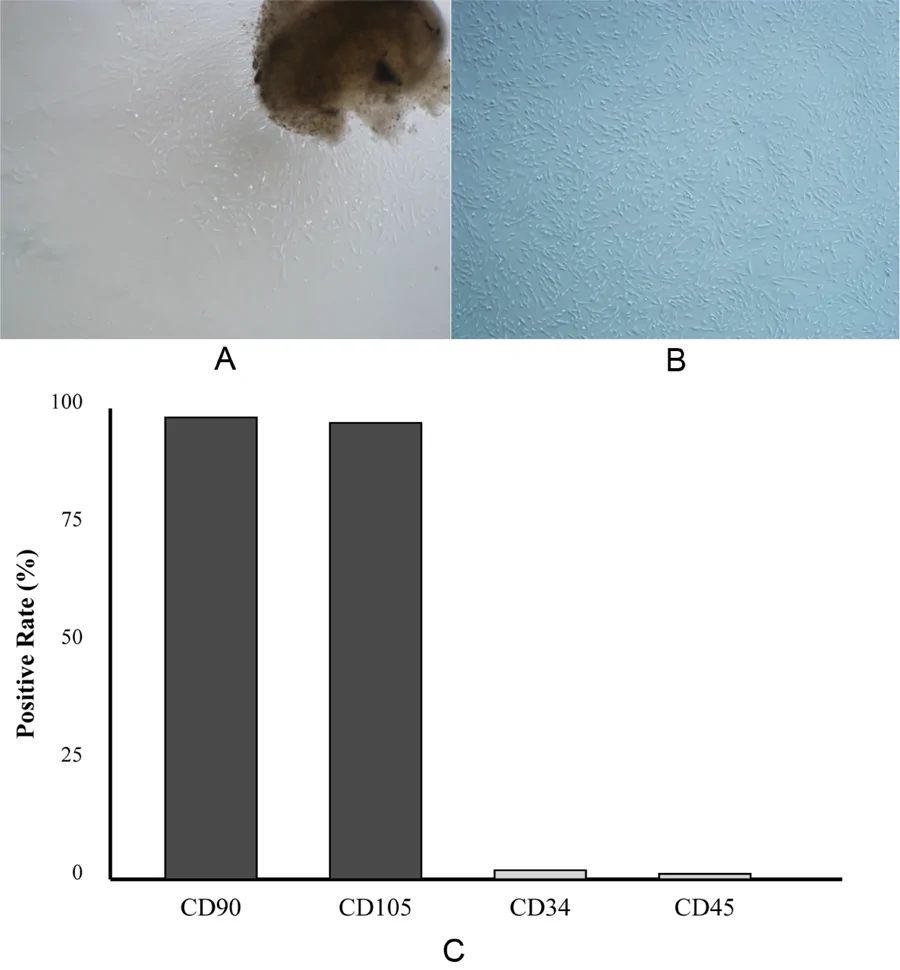
Characterization of human dental pulp stem cells (hDPSCs).

(A) Primary cells migrating from pulp explants showed spindle-shaped morphology (original magnification, ×40). (B) Passage 3 hDPSCs showed a homogeneous fibroblast-like morphology (original magnification, ×100). (C) Flow cytometry: CD90^+^ (98.5%), CD105^+^ (97.8%), CD34^−^ (1.2%), and CD45^−^ (0.8%).

### Optimization of dexamethasone and LPS concentrations

The cytotoxic effects of Dex and LPS were evaluated using the CCK-8 assay. Dex did not significantly affect hDPSC viability at concentrations of 10^9^-10^7^ mol/L (p > 0.05; Figure 2A), whereas 10^6^ and 10^5^ mol/L significantly reduced viability (p < 0.05). A concentration of 10^8^ mol/L, at which viability remained approximately 100% of the control value, was therefore selected for subsequent experiments. LPS at 5 and 10 μg/mL significantly reduced cell viability (p < 0.01; Figure 2B). By contrast, 2 μg/mL LPS produced only a modest reduction in viability (approximately 90% of the control value) while generating sufficient inflammatory stress without extensive cell death. This concentration was therefore selected for the inflammatory model.

**Figure 2:**
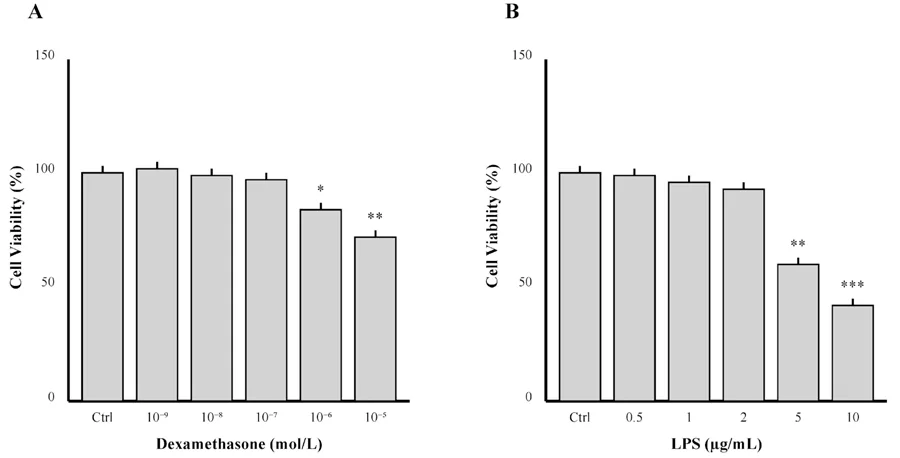
Cytotoxicity assessment of dexamethasone (Dex) and lipopolysaccharide (LPS).

(A) Dex treatment (10^−9^-10^−5^ mol/L, 24 h). (B) LPS treatment (0.5-10 μg/mL, 24 h). Cell viability was measured by CCK-8 assay. Mean ± SD (n = 6). *P < 0.05, **p < 0.01, and ***p < 0.001 vs. control.

### Dexamethasone attenuates LPS-induced proinflammatory cytokine expression and secretion

The anti-inflammatory effect of Dex was evaluated by measuring IL-6, TNF-α, and IL-1β. qRT-PCR showed that LPS markedly increased the mRNA expression of all three cytokines relative to the control group (p < 0.001; Figure 3), whereas Dex pretreatment significantly attenuated these increases (p < 0.001). Cytokine secretion was then assessed by enzyme-linked immunosorbent assay (Figure 4). Consistent with the mRNA findings, LPS substantially increased the concentrations of IL-6, TNF-α, and IL-1β in culture supernatants, and these increases were significantly reduced by Dex treatment (p < 0.01). These findings demonstrate that Dex attenuated the inflammatory response of hDPSCs to LPS.

**Figure 3:**
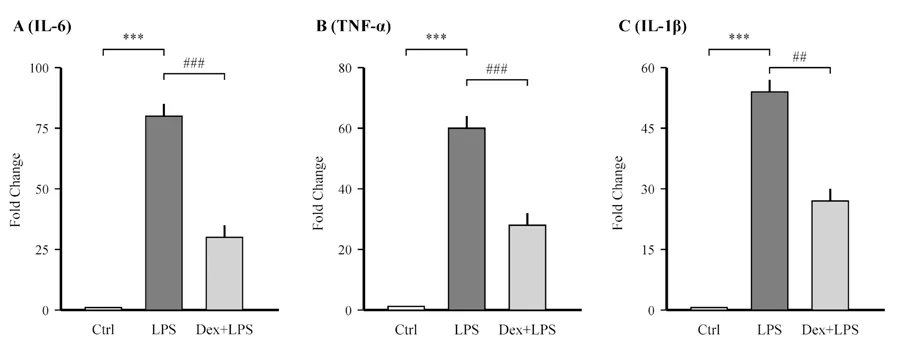
Effect of Dex on proinflammatory cytokine mRNA expression.

Relative mRNA expression of (A) IL-6, (B) TNF-α, and (C) IL-1β was quantified by qRT-PCR. Dex (10^−8^ mol/L) attenuated LPS-induced upregulation. Mean ± SD (*n* = 3). ***p < 0.001 vs. control; ##p < 0.01 and ###p < 0.001 vs. LPS.

**Figure 4:**
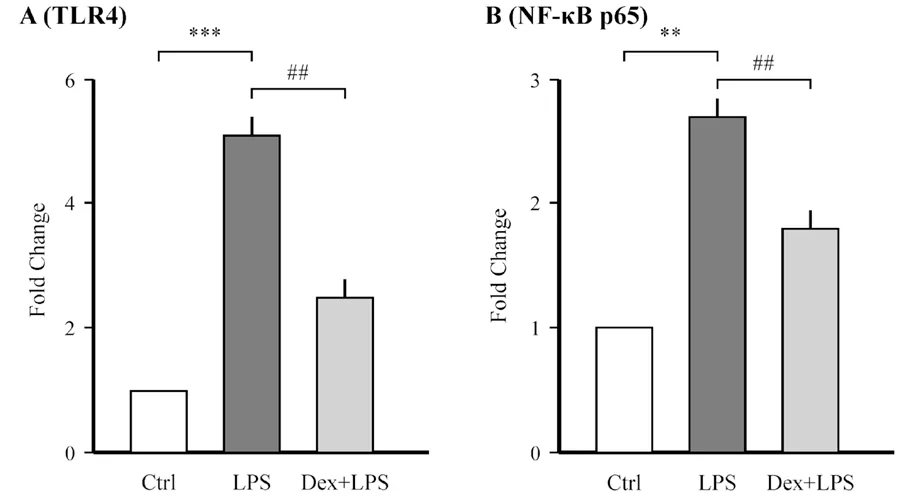
Effect of Dex on TLR4/NF-κB pathway-related gene expression.

Relative mRNA expression of (A) *TLR4* and (B) *NFKB1* was measured by qRT-PCR. Dex attenuated the LPS-induced increases. Mean ± SD (*n* = 3). **p < 0.01 and ***p < 0.001 vs. control; ##p < 0.01 vs. LPS.

### Dexamethasone downregulates TLR4 expression and inhibits NF-κB p65 phosphorylation

To investigate signaling changes associated with the anti-inflammatory effect of Dex, we examined the TLR4/NF-κB pathway. qRT-PCR showed that LPS significantly increased *TLR4* and *NFKB1* mRNA expression, whereas Dex reduced both responses (Figure 5). At the protein level, LPS increased TLR4 abundance and markedly elevated the ratio of phosphorylated p65 to total p65 (p-p65/p65; Figure 6). Dex significantly reduced TLR4 protein expression and p65 phosphorylation (p < 0.01). Densitometric analysis also confirmed that LPS increased IL-1β protein abundance and that Dex attenuated this increase.

**Figure 5:**
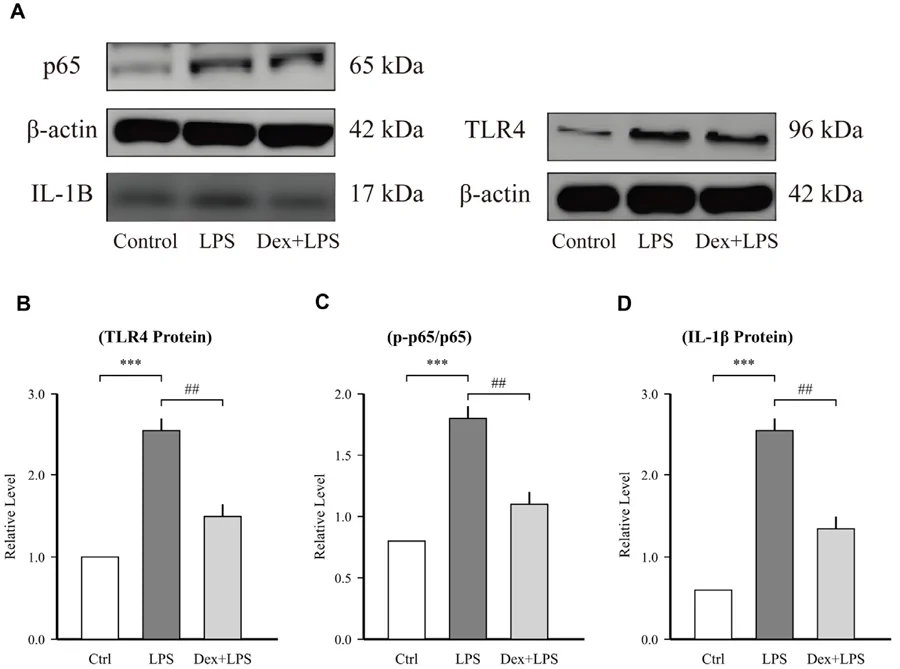
Effect of Dex on TLR4 and NF-κB protein expression.

(A) Representative western blots. Densitometric quantification of (B) TLR4, (C) the p-p65/p65 ratio, and (D) IL-1β. Dex reduced TLR4 expression, p65 phosphorylation, and IL-1β abundance. Mean ± SD (*n* = 3). ***P < 0.001 vs. control; ##*P* < 0.01 vs. LPS.

**Figure 6:**
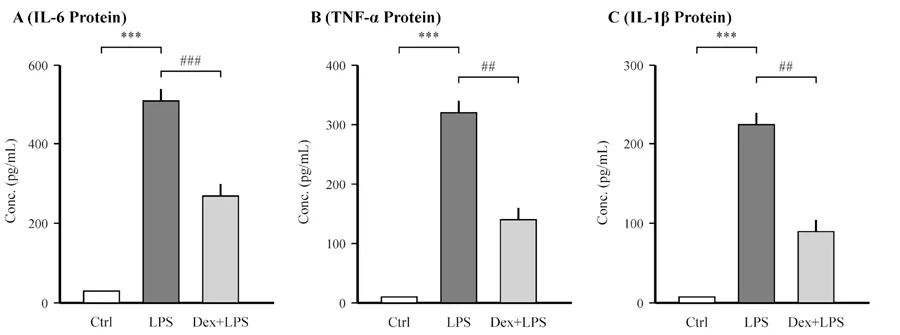
Effect of Dex on proinflammatory cytokine secretion.

ELISA quantification of (A) IL-6, (B) TNF-α, and (C) IL-1β in cell-culture supernatants. Dex reduced secretion of all three cytokines. Mean ± SD (n = 3). ***p < 0.001 vs. control; ##*P* < 0.01 and ###p < 0.001 vs. LPS.

### Dexamethasone restores LPS-impaired odontogenic differentiation

To determine whether Dex restored hDPSC function under inflammatory conditions, we assessed ALP activity and mineralization. LPS significantly reduced ALP activity relative to the control group (p < 0.001), indicating impairment of early odontogenic differentiation. Dex treatment significantly restored ALP activity in LPS-exposed cells (Figure 7A). Similarly, quantitative analysis of Alizarin Red S staining showed that LPS reduced mineralized nodule formation, whereas Dex significantly restored mineralization (Figure 7B). Thus, in addition to suppressing inflammation, Dex preserved the odontogenic differentiation capacity of hDPSCs under LPS-induced inflammatory conditions.

**Figure 7:**
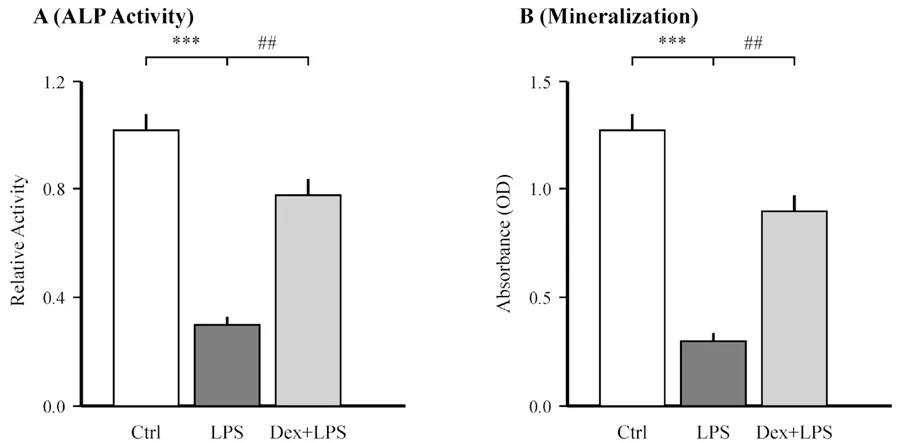
Dex restores LPS-impaired odontogenic differentiation.

(A) ALP activity after 7 days of induction. (B) Alizarin Red S quantification of mineralization after 21 days. Dex restored ALP activity and mineralization suppressed by LPS. Mean ± SD (*n* = 3). ***P < 0.001 vs. control; ##*P* < 0.01 vs. LPS.

Dex alone (10^8^ mol/L) did not significantly alter inflammatory cytokine expression, ALP activity, or mineralized nodule formation under noninflammatory conditions (Table 2), indicating that this concentration had no detectable effect on the measured basal cellular parameters.

**Table 2:** Effects of Dex alone (10−8 mol/L) on basal inflammatory cytokine expression and odontogenic markers in hDPSCs (mean ± SD, n = 3).

Experimental parameter	Control group	Dex-alone group	p- value (t test)
IL-6 mRNA (relative fold change)	1.00 ± 0.05	0.98 ± 0.06	0.685 (n.s.)
TNF-α mRNA (relative fold change)	1.00 ± 0.04	1.02 ± 0.05	0.622 (n.s.)
IL-1β mRNA (relative fold change)	1.00 ± 0.07	0.97 ± 0.05	0.573 (n.s.)
ALP activity (relative value, day 7)	1.02 ± 0.06	1.04 ± 0.05	0.680 (n.s.)
Mineralization (absorbance at 562 nm, day 21)	1.28 ± 0.05	1.31 ± 0.06	0.548 (n.s.)

n.s.: not significant. No significant differences were detected between the untreated control and Dex-alone groups using a two-sided unpaired Student *t* test (all *P* > 0.05). These data indicate that 10^−8^ mol/L Dex alone did not measurably alter the assessed basal inflammatory or differentiation parameters.

## Discussion

The increasing emphasis on VPT has created a need for therapeutic agents that can control inflammation while supporting regeneration.^25^ Although glucocorticoids such as Dex are well-established anti-inflammatory agents, their ability to restore hDPSC function in an inflammatory microenvironment remains incompletely defined. In this study, Dex suppressed LPS-induced inflammatory mediator production in association with inhibition of TLR4/NF-κB signaling and, importantly, restored odontogenic differentiation. These findings indicate a dual anti-inflammatory and pro-regenerative effect and provide a rationale for further preclinical evaluation of localized Dex delivery in regenerative endodontics. This interpretation is also consistent with evidence supporting the clinical anti-inflammatory effects of dexamethasone in symptomatic pulp conditions.^26,27^

We established a viable-cell inflammatory model using 2 μg/mL LPS, which increased IL-6, TNF-α, and IL-1β without causing extensive cell death. This model approximates an inflamed but viable pulp-cell environment. Although endotoxin concentrations measured clinically are generally lower than those used in monolayer culture, microgram-per-milliliter LPS concentrations are commonly required *in vitro* to generate a reproducible TLR4-mediated response. The selected concentration therefore provided a robust, nonlethal inflammatory challenge for evaluating therapeutic rescue. Consistent with previous findings, LPS impaired hDPSC differentiation, as reflected by reduced ALP activity and mineralized nodule formation. This effect may result from both the metabolic demands of the inflammatory response and direct cytokine-mediated inhibition of differentiation pathways. The ability of Dex to reverse these changes is therefore a key finding. By reducing NF-κB-associated inflammatory signaling,^19,21^ Dex may permit reactivation of differentiation-associated programs. This interpretation is consistent with the broader concept that resolution of inflammation supports tissue regeneration and odontogenic/osteogenic differentiation.^28,29^

The pretreatment/co-treatment design demonstrated the protective effect of Dex during LPS exposure; however, clinical pulpitis is typically established before treatment. Co-treatment may partially approximate direct pulp capping or pulpotomy, in which a therapeutic material is placed in contact with exposed, inflamed pulp and must simultaneously attenuate ongoing inflammatory stimuli and support repair. Nevertheless, whether Dex can reverse a fully established inflammatory phenotype remains uncertain. Glucocorticoids can transrepress preactivated NF-κB complexes in other cell types, suggesting that post-exposure treatment may also be effective. The absence of a sequential post-treatment condition, in which LPS stimulation precedes Dex administration, is therefore an important limitation. Future studies should directly compare pretreatment, co-treatment, and post-treatment protocols to strengthen the translational relevance of these findings.

The TLR4/NF-κB axis was strongly associated with the effects of Dex in hDPSCs. LPS increased TLR4 expression and p65 phosphorylation, both of which are characteristic features of LPS signaling.^30,31^ Dex reduced TLR4 abundance and inhibited p65 phosphorylation, suggesting coordinated effects on both receptor-proximal and downstream signaling. Because NF-κB regulates a broad inflammatory transcriptional program, its inhibition is consistent with the reduction in cytokine production observed in the enzyme-linked immunosorbent assays and with the established anti-inflammatory actions of dexamethasone.^32^ Crosstalk between NF-κB and Wnt/β-catenin or bone morphogenetic protein signaling may also be relevant because these pathways contribute to hDPSC differentiation.^33^ Suppression of NF-κB may therefore relieve inflammation-associated inhibition of odontogenic programs. The proposed model is summarized in Figure 8: LPS activates TLR4-dependent p65 phosphorylation and nuclear translocation, increasing cytokine production and suppressing odontogenic differentiation, whereas Dex attenuates this signaling response and restores regenerative function.

**Figure 8:**
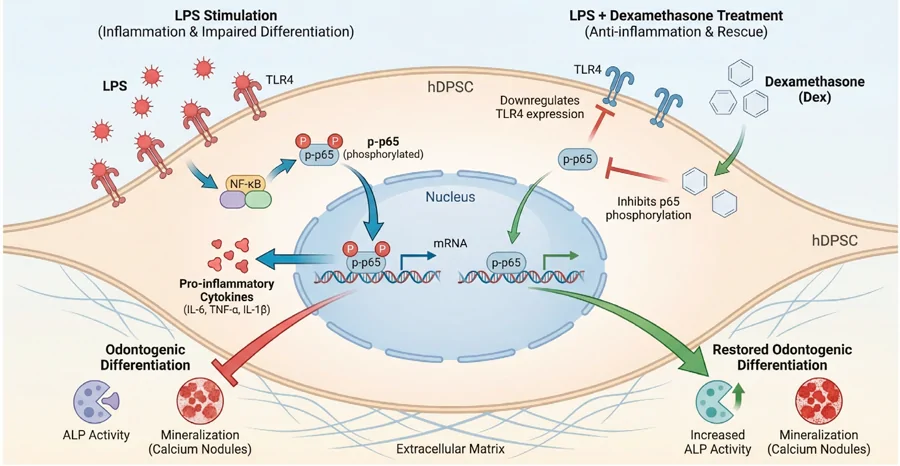
Schematic representation of the proposed mechanism.

LPS activates TLR4/NF-κB p65 signaling, increasing cytokine production and suppressing odontogenic differentiation. Dex reduces TLR4 expression and p65 phosphorylation, thereby attenuating inflammation and restoring hDPSC regenerative capacity.

The effects of Dex were concentration dependent. Cell viability declined at concentrations of 10^6^ mol/L or higher, consistent with the cytotoxic and antiosteogenic effects reported for high-dose glucocorticoid exposure.^34^ By contrast, 10^8^ mol/L Dex attenuated inflammatory signaling without reducing hDPSC viability. These findings support evaluation of carefully controlled, low-dose Dex delivery within pulp-capping materials, where localized exposure may reduce inflammation while preserving reparative capacity.^35^

This study has several limitations. First, the monolayer *in vitro* model does not reproduce the three-dimensional architecture, multicellular interactions, vascular supply, or immune environment of dental pulp. Second, signaling pathways other than TLR4/NF-κB, including MAPK and PI3K/Akt, may contribute to the effects of Dex; transcriptomic studies have identified enrichment of MAPK-related genes in Dex-treated dental pulp cells.^20^ Third, the study did not include pathway-specific pharmacological inhibitors, such as the TLR4 inhibitor TAK-242 or the NF-κB inhibitor BAY 11-7082, or gene-silencing experiments. The observed changes therefore support an association with TLR4/NF-κB signaling but do not establish pathway-specific causality. Future studies should incorporate mechanistic perturbation, three-dimensional organoid systems, sequential treatment designs, and *in vivo* pulpitis models to verify the pathway and evaluate long-term reparative dentin formation.

## Conclusion

In this *in vitro* model, Dex attenuated LPS-induced inflammation and restored the odontogenic differentiation capacity of hDPSCs. These effects were accompanied by reduced TLR4 expression and NF-κB p65 phosphorylation. The findings provide a molecular and functional basis for further investigation of localized, low-dose Dex delivery as an adjunct to vital pulp therapy.

## Data Availability

The datasets used and/or analyzed during the current study are available from the corresponding author upon reasonable request.
